# Mapping evidence on adolescents’ HIV-positive status disclosure in sub-Saharan Africa: a protocol for a scoping review

**DOI:** 10.1186/s13643-020-01546-9

**Published:** 2020-12-04

**Authors:** Patience Adzordor, Clement Avoka, Vitalis Bawontuo, Silas Agbesi, Desmond Kuupiel

**Affiliations:** 1 Faculty of Health and Allied Sciences, Catholic University College of Ghana, Fiapre, Sunyani, Ghana; 2 Research for Sustainable Development (r4ds) Consult, Sunyani, Ghana; 3 School of Medicine, University of Health and Allied Sciences, Ho, Ghana; 4 Department of Public Health Medicine, School of Nursing and Public Health, University of KwaZulu-Natal, Durban, 4001 South Africa

**Keywords:** Adolescents, HIV/AIDS, Sero-positive status, Disclosure, Sub-Saharan Africa, Scoping review

## Abstract

**Background:**

Sub-Saharan Africa (SSA) homes most of the people living with HIV/AIDS in the world. Adolescents/young people are a vulnerable population and at high risk of HIV infection. Identifying and bridging the research gaps on the disclosure of HIV-positive status among adolescents, particularly to their sexual partners, is essential to inform appropriate policy planning and implementation towards preventing HIV transmission. This study will aim to explore literature and describe the evidence on HIV-positive status disclosure among adolescents in SSA.

**Methods:**

The framework provided by Arksey and O’Malley’s framework and improved by Levac and colleagues will be used to conduct a scoping review. A keyword search for relevant literature presenting evidence on HIV-positive status disclosure among adolescents in SSA will be conducted in CINAHL, PubMed, Science Direct, Google Scholar, and SCOPUS. Date limitations will be removed, but Boolean terms “AND” and “OR” as well as Medical Subject Headings terms will be included where possible and syntax modified to suit the database during the search. Additional relevant articles will be sought from the reference lists of all included studies using a snowballing method. Two reviewers will independently screen the articles at the abstract and full-text screening phases in order to reduce bias and improve the reliability of this study’s findings. A tabular form will be developed using Microsoft Word and piloted for data extraction. Thematic content analysis will be conducted, and a narrative summary of all relevant outcomes reported. Quality appraisal of the included studies for this proposed study will be performed utilizing the recent mixed methods appraisal tool.

**Discussion:**

The evidence produced by this review may help inform policy and strategies to reduce the incidence of HIV infection among adolescents and improve social support for adolescents living with HIV/AIDS in SSA. It may also reveal literature gaps to guide future researches to further inform HIV policies for adolescents in SSA. Platforms such as peer review journals, policy briefs, and conferences will be used to disseminate this study’s findings.

**Supplementary Information:**

The online version contains supplementary material available at 10.1186/s13643-020-01546-9.

## Background

The burden of human immunodeficiency virus (HIV) and acquired immune deficiency syndrome (AIDS) remains a public health concern globally, particularly in sub-Saharan Africa (SSA). Evidence shows that SSA is home to about 90% of the world’s HIV/AIDS burden [1, 2]. Adolescents, youth, and young people are a vulnerable population and are at high risk of HIV infection owing to several reasons including peer influences and high rates of unprotected sex [3]. The World Health Organization (WHO) defines “Adolescents” as individuals in the 10–19-year age group and “Youth” as the 15–24-year age group, while “Young People” covers the age range of 10–24 years. In 2018, the UNAIDS global estimates showed that about 5000 new HIV infections occur each day among adolescents and children of which close to 66% occur in SSA [1]. Of these 5000 daily new cases of HIV, about 600 occur in populations age less than 15 years, and about 4400 occur among people aged 15–24 years (of whom almost 43% are among young women [1].

Despite this, research has shown that adolescents have low rates of disclosure of their HIV-positive status to particularly their sexual partners [4, 5]. Reasons for HIV-positive status non-disclosure might be multifaceted. A study revealed that negotiating safe sexual practices is particularly challenging for HIV-positive adolescents, exacerbated by HIV-related factors such as learning and accepting their status and stigmatization, and they may withhold or fail to disclose their HIV-status to sexual partners [3]. It is evident that HIV status disclosure among adolescents may be influenced by several factors including knowing one’s partner’s status, stigma, medication taking, desire for love and acceptance, and progression of HIV disease [3–7]. Nonetheless, HIV status disclosure among other benefits is essential to ensure safer sex practice and prevent the transmission of the virus from an infected sexual partner to the other according to the WHO [8]. In addition to the public health benefits of disclosure that include an expanded awareness of the risk that may lead to decreased sexual risk-taking and ultimately decreased transmission of HIV, there are also potential benefits to the individual who chooses to share results with his/her sexual partners [8]. Disclosure of HIV status to sexual partners, for instance, may lead to increased opportunities for instrumental and expressive social support; improved access to necessary medical treatment and care; increased opportunities to discuss and implement HIV risk reduction with partners; and increased opportunities to plan for the future carefully and thoughtfully [8]. To this end, the WHO recommends disclosure to adolescents of their own HIV status before counselling on potential benefits and risks of disclosure to others [8].

Although a previous systematic review revealed a paucity of research on associations between disclosure and negotiating safe sexual practices [9], to date, the evidence before this study shows that no study has comprehensively examined literature and mapped evidence on HIV-positive status disclosure among adolescents. Therefore, this study will aim to map literature and describe the evidence on HIV-positive status disclosure among adolescents in SSA using a scoping review methodology. This study will focus on the prevalence of disclosure; person(s) adolescents living with HIV/AIDS disclose their status to; predictors of HIV-positive status disclosure/non-disclosure; and the relation between safe sex practice and HIV-positive status disclosure among adolescents in SSA. The evidence produced by this review may help inform policy and strategies to reduce the incidence of HIV infection among adolescents as well as improve social support for adolescents living with HIV/AIDS in SSA. This study potentially will also reveal literature gaps to guide future researches to further inform HIV policies for adolescents in SSA. Also, this study may contribute towards the attainment of the United Nations Sustainable Goal 3.3 which stipulates the ending of AIDS epidemic by 2030. Moreover, this study will add to the existing literature on HIV-positive status disclosure among adolescents in SSA.

## Methods

The Preferred Reporting Items for Systematic Reviews and Meta-Analyses guideline for study Protocols (PRISMA-P) was adopted to develop this protocol (Supplementary file 1). This study will employ a scoping review methodology to answer the research question. This is because scoping reviews are considered useful in representing a series of literature that exists around a subject of interest and helps to focus the research questions by registering existing research findings and identifying research gaps [10]. A scoping methodology is also considered a useful approach for determining the basic and value of follow-up of a primary study or a full systematic review [10]. This proposed scoping review study will be guided by the improved version of Arksey and O’Malley’s framework [11]. The steps outlined in Arksey and O’Malley’s framework [11] are as follows: identifying the research question, identifying relevant studies, study selection, charting the data, and collating, summarizing, and reporting results.

### Identifying the research question

The main research question of this review will be as follows: To date, what is the evidence on adolescents’/young people’s HIV-positive status disclosure in SSA? The sub-research questions for this proposed review are as follows:

1. What evidence exists on the prevalence of HIV-positive status disclosure to and by adolescents/young people in SSA?
2. What evidence exists on who adolescents/young people disclose their HIV-positive status to in SSA?
3. What evidence exists on the predictors of HIV-positive status disclosure/non-disclosure among adolescents/young people in SSA?
4. Is there evidence on the relationship between safe sex practice and HIV-positive status disclosure among adolescents/young people in SSA?

The Population, Concept, and Context (PCC) framework used to determine the eligibility of the primary research question for the proposed scoping review is shown in Table 1.

**Table 1:** The PCC defining the suitability of the scoping review question

Population	This study’s population will include individuals aged between 10 and 24 years old.
Concept	Disclosure of HIV status: Knowing your HIV status, informing someone about it such as sexual partner(s), family member(s), and others.
Context	Prevalence of disclosure to/by adolescents/young people, who adolescents/young people disclose to, predictors of disclosure, safe sex practice in SSA countries: This will include all countries listed among the World Health Organization Africa Region (Algeria, Angola, Benin, Botswana, Burkina Faso, Burundi, Cabo Verde, Cameroon, Central African Republic, Chad, Comoros, Congo, Cote d’Ivoire, Democratic Republic of Congo, Equatorial Guinea, Eritrea, Eswatini, Ethiopia, Gabon, Gambia, Ghana, Guinea, Guinea Bissau, Kenya, Lesotho, Liberia, Madagascar, Malawi, Mali, Mauritania, Mauritius, Mozambique, Namibia, Niger, Nigeria, Rwanda, Sao Tome and Principe, Senegal, Seychelles, Sierra Leone, South Africa, South Sudan, Togo, Uganda, United Republic of Tanzania, Zambia, and Zimbabwe)

### Identifying relevant studies

We will conduct a thorough search in the following electronic databases: PubMed, Science Direct, CINAHL, SCOPUS, and Google Scholar for relevant articles published from 2000 through to the last search date in 2020. A comprehensive search strategy will be developed in consultation with an experienced librarian using keywords such as “adolescent” OR “young people” OR “teenager” OR “youth” AND “HIV” OR “hiv” OR “human immunodeficiency virus” OR “AIDS” OR “hiv/aids” OR “acquired immune deficiency syndrome” AND “status disclosure” OR “sero-status disclosure” OR “disclosure”. Medical Subject Heading (MeSH) terms and subject heading will be included and modified appropriately to suit each database during the electronic database search. Limitations on study designs, year of publication, and publication language will be removed during the search to facilitate capturing as many as possible, all relevant articles to answer the review question. Table 2 shows a pilot search for literature conducted in PubMed demonstrating the possibility of conducting this proposed scoping review study. We will also manually explore the reference lists of all included studies for additional relevant studies utilizing a snowball method. Each search will be documented appropriately, and Mendeley Desktop use to compile and manage all references.

**Table 2:** A pilot search conducted in the PubMed database for this review

Date	Database	Keywords	Search results
8/06/2020	PubMed	((((((((“adolescent”[MeSH Terms] OR “adolescent”[All Fields]) AND (“young”[All Fields] AND (“persons”[MeSH Terms] OR “persons”[All Fields] OR “people”[All Fields]))) OR (“adolescent”[MeSH Terms] OR “adolescent”[All Fields] OR “teenager”[All Fields])) OR (“adolescent”[MeSH Terms] OR “adolescent”[All Fields] OR “youth”[All Fields])) AND (“hiv”[MeSH Terms] OR “hiv”[All Fields])) OR (“hiv”[MeSH Terms] OR “hiv”[All Fields])) OR (“hiv”[MeSH Terms] OR “hiv”[All Fields] OR (“human”[All Fields] AND “immunodeficiency”[All Fields] AND “virus”[All Fields]) OR “human immunodeficiency virus”[All Fields])) OR (“acquired immunodeficiency syndrome”[MeSH Terms] OR (“acquired”[All Fields] AND “immunodeficiency”[All Fields] AND “syndrome”[All Fields]) OR “acquired immunodeficiency syndrome”[All Fields] OR “hiv aids”[All Fields])) AND (status[All Fields] AND (“disclosure”[MeSH Terms] OR “disclosure”[All Fields]))	28,731

### Eligibility criteria

To ensure the selection of relevant studies for this review, the study selection will be guided by the eligibility criteria as specified under the inclusion/exclusion criteria.

#### Inclusion criteria

This study will include published articles that meet the following criteria:

- Studies involving any country or countries included in the WHO Africa Region
- Studies focused on adolescents aged between 10 and 24 years old/studies with 80% of its study population within 10–24-year age group
- Studies presenting evidence of HIV-positive status disclosure
- Studies reporting findings on prevalence, predictors, persons disclose to, and safe sex relating to HIV-positive status disclosure
- All primary study designs
- Publications in English

#### Exclusion criteria

This study will exclude the following:

- Studies conducted in countries outside the WHO Africa Regions,
- Studies targeting HIV-negative adolescents
- Studies reporting findings on knowledge, attitude, and perception of adolescents toward HIV/AIDS
- Publications in other languages either than English
- Papers not reporting primary research data

#### Study selection

**Fig. 1:**
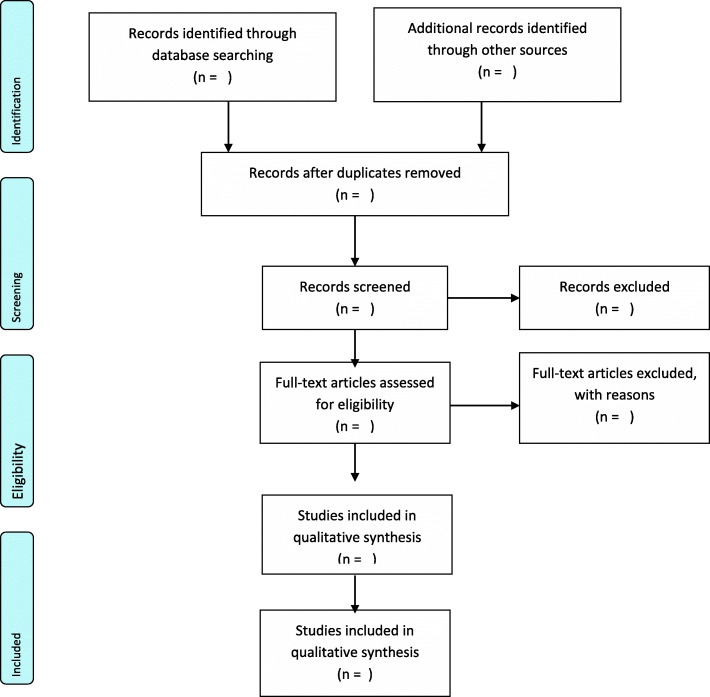
PRISMA flow diagram

First, the principal researcher (AP) will conduct the database search supervised by BV and DK. Guided by this study’s eligibility criteria, all relevant or related titles will be identified and imported onto the Mendeley Desktop Library created for the study. At the second (abstract screening) and third (full-text screening) stages, AP and CA will independently sort the studies into two classifications (“included” and “excluded”) using the eligibility criteria. Discrepancies between AP and CA at the abstract screening stage will be resolved through a discussion by the review team until a consensus is reached. However, DK will independently resolve discrepancies between AP and CA at the full-text screening stage. In the situation where a full-text article cannot be found from the databases, assistance would be sought from the Catholic University’s library or the full-text will be requested from the original authors via email. To ensure trustworthiness, the inter-rater reliability (Cohen’s kappa co-efficient (*k*) statistics) between reviewers’ responses will be calculated using SPSS version 25 following full-text screening. Kappa static less than 50% will be interpreted as poor agreement, 50–70% (moderate agreement), and 70–100% (good agreement). However, we will hope to reach a good agreement. The search records such as the date of search, database, keywords, number of retrievable studies, and number of eligible studies will be adequately documented. To ensure accountability, this study will adopt the PRISMA flow diagram to present the screening results (Fig. 1).

### Charting the data

A data charting form will be developed for the extraction of relevant data from the included studies for this scoping review. Table 3 shows the components of the proposed data extraction form for this study. The data extraction form will be piloted by two independent reviewers (AP and SA) using a random sample of 10% of the included studies to ensure consistency and accuracy. The data extraction form will be adjusted as required based on feedback from the two independent reviewers. We will constantly update the data extraction form to enable adequate abstraction of all relevant data to answer the review question.

**Table 3:** Proposed data extraction form

Author	
Date of publication	
Study of objective	
Type of study design	
Country	
Study setting	
Study population	
Number of study participants	
Sample size	
Number with known HIV infection	
Number disclosed	
Person disclosed to	
Reasons for non-disclosure	
Factors influencing disclosure	
Relationships between safe sex and HIV-positive status disclosure	
Other significant findings	
Conclusions and recommendations	

### Quality appraisal

To evaluate the quality of the studies that will be included in this proposed review, the Mixed Method Quality Appraisal Tool (MMAT) version 2018 will be used to evaluate the methodological quality of all the included primary study designs (randomized control trials, non-randomized control studies, quantitative descriptive studies, quality studies, and mix-methods studies) [12]. The relevance of the study, study design, adequacy and methodology, data collection, analysis of data, and study findings will be examined using the MMAT tool. The quality assessment will help report the risk of bias of the studies included. The quality of the included studies will be graded by calculating the total percentage quality score as specified by the 2018 MMAT. A percentage quality score ranging from ≤ 50% will be considered as low quality, 51–75% will be considered as an average quality, and 76–100% will be considered as high quality. To reduce/address bias, the quality appraisal will be conducted by two reviewers. Any discrepancies between the reviewers will be discussed by the review team until a consensus is reached.

### Collating, summarizing, and reporting results

Thematic content analysis [13] will be conducted following the data extraction. This will help to identify all relevant findings and themes to address the research question. The findings will be structured around the following themes/outcomes: prevalence of HIV-positive status disclosure, predictors of HIV status disclosure, person(s) an adolescent disclose their HIV-positive status to, reasons of non-disclosure, and HIV-positive status disclosure and safe sex practice. This study will additionally analyse all other relevant emerging themes. The sub-themes will be collated, summarized, and a narrative account of the findings reported for each theme or outcome. Tables and graphs will also be used to represent findings where possible. Moreover, this study will explore the possibility of conducting a meta-analysis using quantitative data obtained from this scoping review.

## Discussion

The disclosure of one’s HIV-positive status to the partner plays a crucial role in the prevention and reduction of the incidence of HIV/AIDS. People living with HIV not only struggle with an incurable disease but also go through a lot of psychological trauma as to whether to make their partners aware of their status. Despite the numerous public health benefits that may be achieved as a result of HIV-positive status disclosure to partners, there are series of potential risks such as loss of economic and social support, abandonment, blame, physical and emotional abuse, discrimination, and disruption of family relations [14]. These risks may hinder HIV-positive clients from disclosing their status to their partners. Therefore, there is the need for HIV-positive clients to understand the benefits of disclosing their HIV status to their partners in order to reduce the spread of the infection, promote access to healthcare, enhance mental and emotional support from relatives and friends, reduce stigma, enhance treatment adherence, and promote healthy behaviour [15].

It is anticipated that the results of the proposed study will inform future research and reveal evidence-based information to address HIV-positive disclosure among adolescents to their sexual partners in SSA. The proposed study will also be useful to other SSA countries planning to implement some health policy, as they will be able to draw useful lessons to guide them through the process. The proposed study will also help inform policymakers in updating and implementing protocols to address key issues especially on stigmatization that prevents disclosure of HIV status among adolescents who tested positive.

## Supplementary Information

**Additional file 1.** PRISMA-P 2015 Checklist.

## Data Availability

We have duly cited all studies and data is presented in a form of references.

## Abbreviations

AIDS: Acquired immune deficiency syndrome
HIV: Human immunodeficiency virus
MMAT: Mixed Method Quality Appraisal Tool
SSA: Sub-Saharan Africa
WHO: World Health Organization
